# Case Report: A Rare Truncating Variant of the *CFHR5* Gene in IgA Nephropathy

**DOI:** 10.3389/fgene.2021.529236

**Published:** 2021-05-20

**Authors:** Gabriella Guzzo, Salima Sadallah, Heidi Fodstad, Jean-Pierre Venetz, Samuel Rotman, Daniel Teta, Thierry Gauthier, Giuseppe Pantaleo, Andrea Superti-Furga, Manuel Pascual

**Affiliations:** ^1^Organ Transplant Center, Lausanne University Hospital, University of Lausanne, Lausanne, Switzerland; ^2^Service of Immunology and Allergy, Lausanne University Hospital, University of Lausanne, Lausanne, Switzerland; ^3^Service of Nephrology, Valais Hospital, Sion, Switzerland; ^4^Division of Genetic Medicine, Lausanne University Hospital, University of Lausanne, Lausanne, Switzerland; ^5^Service of Clinical Pathology, Lausanne University Hospital, University of Lausanne, Lausanne, Switzerland; ^6^Hôpital Riviera Chablais, Vevey, Switzerland

**Keywords:** IgA nephropathy, complement, *CFHR5*, CFH, eculizumab, atypical hemolytic uremic syndrome, thrombotic microangiopathy (TA-TMA)

## Abstract

IgA nephropathy (IgAN) is the most common primary glomerulonephritis worldwide. Despite appropriate therapy, 20–40% of affected-patients evolve toward end-stage kidney disease (ESKD). Mesangial IgA deposits are the hallmark of IgAN, and complement deposition (C3) seems to differentiate latent IgA mesangial deposits from active IgAN. Atypical hemolytic uremic syndrome (aHUS), another disease in which complement plays an important role, is caused by inherited or acquired deregulation of the alternative pathway (AP) of complement. A subgroup of IgAN shows thrombotic microangiopathy (TMA) lesions in kidney biopsies, the histological characteristic of aHUS. Genetic variants of complement Factor H (CFH), known to be present in aHUS, have been associated with rapidly progressive forms of IgAN and a clinical pattern of aHUS. Genome-wide association studies (GWAS) have confirmed that the 1q32 region, encoding for *CFH* and its related proteins, is an IgAN susceptibility locus. A 30 year-old man was admitted for seizures and malignant hypertension. The kidney biopsy showed IgAN associated with features of TMA. Despite five plasma exchanges, the patient remained dialysis-dependent, and ESKD was diagnosed. Functional and genetic complement analysis were performed. A monoallelic protein-truncating, likely loss-of-function variant was identified in the *CFHR5* gene. Eculizumab is the treatment of aHUS. As it has been successfully used in a few cases of rapidly progressive IgAN, it was decided to administer eculizumab over a period of 12 months in addition to the usual immunosuppression for renal transplantation. After a follow-up of 3 years, there was no clinical disease recurrence. Systematic biologic and genetic screening of complement in individuals with IgAN might be useful to better delineate the role of the AP of complement in renal disease progression, and this may have therapeutic implications.

## Introduction

IgA nephropathy (IgAN) is the most common cause of end-stage kidney disease (ESKD) among primary glomerulonephritis (Donadio and Grande, 2002). Even though IgAN was first described more than half a century ago by Berger et al. the pathogenesis remains incompletely elucidated (Berger and Hinglais, 1968).

The hallmark of IgAN is the mesangial deposition of IgA1 immunoglobulins with an aberrant glycosylation (Moldoveanu et al., 2007). While over 50% of patients with IgAN have increased IgA1 serum level compared to healthy controls, this alone is not sufficient to cause the disease. The mesangial accumulation of IgA1 has been described as the first step of a “4-hit process,” which finally leads to complement activation (Suzuki et al., 2011). The second hit is the occurrence of IgG or IgA1 autoantibodies directed against the antigenic galactose deficient IgA1 (Gd-IgA1). Formation of circulating immune complexes (CIC) containing Gd-IgA1 then occur (third hit) and their mesangial deposition (fourth hit) triggers cells hyperplasia and hypertrophy, release of inflammatory cytokines and chemokines with complement activation. The frequent post-transplant disease recurrence confirmed the CIC mediated nature of IgAN (Ortiz et al., 2012). Conversely, IgA deposits are rapidly cleared from kidneys with donor-derived IgAN when transplanted in recipients without IgAN as cause of ESKD (Sanfilippo et al., 1982; Ponticelli et al., 2001).

The co-localization of C3 and IgA deposits in 90% of IgAN kidney biopsies indicates complement activation by Gd-IgA1. Therefore, complement plays a key role in the pathogenesis of IgAN (Maillard et al., 2015). IgA mesangial deposition mostly occurs in active kidney disease (Suzuki et al., 2003), and C3 co-deposition may be considered as a biomarker of nephritogenic IgA1 deposition, where local and systemic evidence of complement activation are prognostic marker of IgAN (Hastings et al., 2013).

Clinical and pathological manifestations of IgAN can vary widely, with a 20–40% subgroup evolving to ESKD. The identification of clinical, biological and/or histological prognostic markers at diagnosis could thus help tailoring the best treatment option to the given patient's risk profile (Barbour et al., 2019).

Complement in IgAN is activated by both the alternative pathway (AP) and the lectin pathway (LP), as demonstrated by the variable immunohistochemical positivity for properdin, complement factor H (CFH), complement FH related proteins (CFHRs), C4d, mannose binding lectin (MBL), MBL-associated serine proteases (MASPs), L-ficolin and C5b-9 (Maillard et al., 2015). The classical pathway of complement does not seem to contribute to the pathogenic process, indeed C1q deposits are rarely found and only confined in sclerotic areas (Lee et al., 2013).

An interesting question is whether dysfunctional regulation of complement activation may have a significant role in the rapid progression to ESKD in some forms of IgAN (Tortajada et al., 2019). Two genome-wide association studies (GWAS) identified and confirmed 1q32 as a locus of IgAN susceptibility (Gharavi et al., 2011; Kiryluk et al., 2012). The 1q32 region codes for CFH, the major regulator of complement in soluble phase, and five CFHRs, which can compete with CFH and deregulate the AP of complement.

Higher plasma CFHR5 protein levels (Zhu et al., 2018) and increased CFHR5 glomerular deposition have been associated with progressive IgAN (Medjeral-Thomas et al., 2017), but no genetic analysis was performed in both studies. A severe form of IgAN was associated with *CFH* mutations and a clinical pattern of atypical hemolytic uremic syndrome (aHUS), and eculizumab was successfully employed in the treatment of this patient (Nakamura et al., 2018).

We report a case of rapidly progressive IgAN associated with a clinical pattern of aHUS and thrombotic microangiopathy (TMA) features on kidney biopsy, leading to ESKD. Genetic complement screening identified CFH-H3, MCP-H2, CFHR1^*^B polymorphisms, known to constitute moderate risk factors for aHUS, together with a rare deleterious variant in the *CFHR5* gene. Treatment with eculizumab successfully prevented clinical recurrence of the disease, with a follow-up of 3 years after renal transplantation.

## Case Report

**Figure 1:**
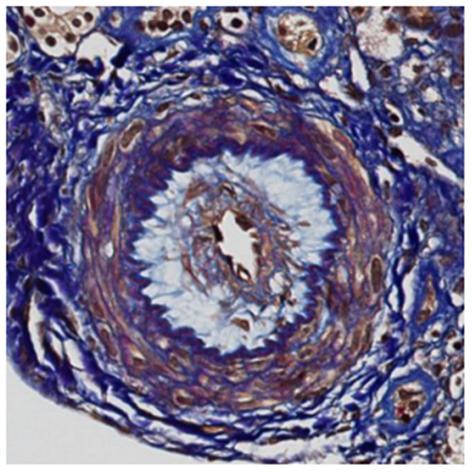
Light microscopy. Early stage of thrombotic microangiopathy: this artery shows oedematous intima and few myointimal cells corresponding to “mucoid intimal hyperplasia” (FAOG, 400x).

**Figure 2:**
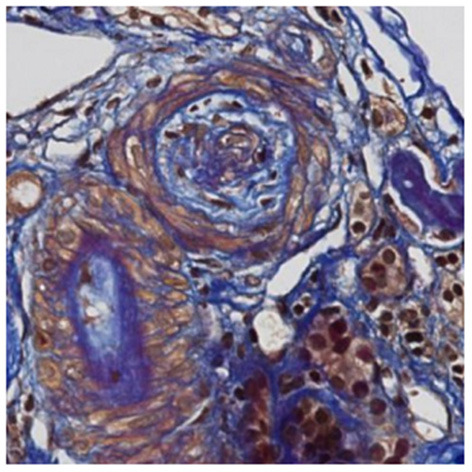
Light microscopy. Later changes of thrombotic microangiopathy: the artery contains fibro-oedema with few collagen fibers within intima revealed in blue with trichome FAOG (FAOG, 400x).

**Figure 3:**
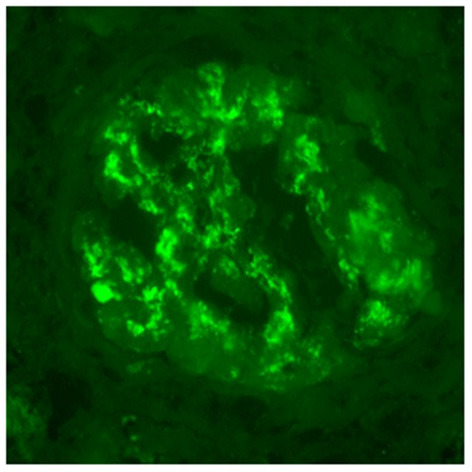
Immunofluorescence microscopy. IgA deposits are observed within mesangium and glomerular membranes (400x).

A 30 year-old Asian man, born in Sri-Lanka, was referred to our hospital for a kidney pre-transplant evaluation. At the age of 16, he presented a unique episode of gross hematuria and lower limb oedema, which was not furtherly investigated. Of note, the patient showed no family history of TMA, IgA nephropathy or other kidney diseases. At the age of 28, he was admitted to the hospital for recurrent generalized seizures and oliguria, as a manifestation of malignant hypertension. Laboratory analysis revealed hemolytic anemia (hemoglobin 71 g/L; schistocytes 9‰, LDH 348 U/L, haptoglobin <0.10 g/L), thrombocytopenia (70 × 10^3^/μL) and severe kidney failure (creatinine 2,013 μmol/l) with proteinuria (2.26 g/mmol from urine protein/creatinine ratio). Kidney biopsy showed diffuse glomerular sclerosis (90%), interstitial fibrosis (90%) and tubular atrophy, associated with severe vascular lesions characteristic of TMA (Figures 1, 2). Immunofluorescence was positive for abundant mesangial IgA (Figure 3) and C3 deposits, with focal IgA staining in some arterioles. In addition, deposits of C5b-9, were present with IgA and C3 deposits, within the mesangium and the wall of small arterioles.

IgAN associated with TMA was diagnosed. Five plasma exchanges were performed without improvement of renal function, and the patient remained dialysis-dependent.

## Complement Assays and Genetic Analysis

One year after starting dialysis, no abnormality was detected in the complement screening (C4, CH50, CFH, Factor I, CD46, or anti-CFH autoantibodies). At the time of pre-transplant evaluation, he had persistently low platelet counts and low C3 level (0.66 g/L; reference range > 0.75 g/L).

Given the suspicion of aHUS, functional evaluation of complement and molecular genetic analysis were performed. We used an in-house genetic panel that we routinely apply at our institutions to individuals with aHUS suspected to have a genetic origin. This panel, that includes a NGS [Next-Generation Sequencing using a TruSight One Expanded Sequencing Panel (Illumina, USA)] analysis of ADAMTS13, C3, CD46, CFB, CFD, CFH, CFHR1, CFHR2, CFHR3, CFHR4, CFHR5, CFI, CFP, HOXA2, MMACHC, THBD, has been assembled by our nephrologists, pediatric nephrologists and geneticists and is quite similar to that used in diagnostic laboratories worldwide.

The patient was found to be a carrier of the haplotypes *CFH-H3* and *MCP-H2* and the CFHR1^*^B polymorphism (homozygous), which are common and have been associated with a slightly increased risk for aHUS (Abarrategui-Garrido et al., 2009). More significantly, he was found to be heterozygous for a very rare *CFHR5* variant (Chr.1: 196953197-TC>T; NM_030787.4, c.361delC, p.Gln121Lysfs^*^10, rs778029757), which leads to a frameshift/premature stop codon [dbSNP Database, 2021; Genome Aggregation Database (gnomAD), 2021]. It predicts the synthesis of a CFHR5 protein with a normal amino acid sequence from the N-terminus up to the SCR 2 domain, followed by a short stretch of 10 missense amino acids and then terminating abruptly, leading to the absence of two-thirds of the protein. Such proteins are frequently unstable. Alternatively, a stop codon occurring in the first third of the mRNA might likely lead to nonsense-mediated mRNA decay (NMD). In both cases, haploinsufficiency would be the consequence. However, in absence of cellular studies, it cannot be formally excluded that the variant may induce a dominant negative effect on CFHR5 function (see section Discussion, below).

The variant is represented in the gnomAD database [Genome Aggregation Database (gnomAD), 2021] with 12 alleles on a total of ~250,000 (allelic frequency 0.000048, or 0.0048%; one individual in ~11,000) and all carriers are of South Asian origin (our patient is from Sri Lanka). It has not been seen at homozygosity and has not been reported in association with IgAN so far. In spite of its deleterious effect on the protein, its classification, according to the ACMG guidelines, is between **variant** of unknown significance or likely benign variant (because of the presence of 12 heterozygous presumably healthy individuals in gnomAD). However, this does not exclude its role as a disease predisposing factor (see below).

## Patient Outcomes

After a pre-transplant work-up, the patient received a kidney allograft from a living unrelated donor. The day of transplant, complement analysis revealed a low CH50 (59%; normal value > 70%) and C3 (0.64 g/L). AP50, MBL, CFH, circulating C5b-9 were normal and there were no CFH autoantibodies.

Crossmatch assays were negative by both complement dependent cytotoxicity (CDC) and flow cytometry techniques. HLA match was of 2/6 antigens (A24, DQ5). Induction and maintenance immunosuppression consisted of basiliximab (20 mg at days 0 and 4), steroids, mycophenolate mofetil (MMF) and tacrolimus. Considering the possible risk of aHUS recurrence associated with the multiple complement genetic abnormalities found, prophylactic eculizumab was administered peri- and post–transplant. Initially he received a weekly dose of eculizumab (600 mg, D1 and D8), followed by one intravenous administration approximately every 3 weeks (900 mg), to maintain a CH50 <10%. No acute rejection occurred. Eculizumab was discontinued after 1 year post-transplant.

So far, an excellent allograft function has been observed, with no clinical evidence of IgA recurrence after transplantation and the patient is currently doing well with a serum creatinine of 92 μmol/l (eGFR by CKD-EPI of 92 ml/min/1.73 m^2^) and no proteinuria.

## Discussion

Over the years, evidence has accumulated on the pathogenic role of complement in IgAN. Already in 1994, Stad et al. showed that complement depletion induced by cobra venom factor abolished glomerular inflammation, proteinuria and C3 deposition in a rat model of IgAN (Stad et al., 1994).

Furthermore, hereditary or acquired deregulation of the AP of C may exacerbate the prognosis of IgAN (Gharavi et al., 2011; Kiryluk et al., 2012; Zhai et al., 2016; Medjeral-Thomas et al., 2017; Nakamura et al., 2018; Zhu et al., 2018; Tortajada et al., 2019). The 1q32 gene locus has been associated with IgAN. It codes for CFH and the five CFH-related (CFHR) proteins (Gharavi et al., 2011; Kiryluk et al., 2012). These proteins have a high degree of gene sequence identity, particularly in the C-terminal regions, which together with the gene locus proximity favor non-homologous recombination and gene rearrangements. Abnormal CFHRs may compete with CFH, interfering with its inhibitory activity and potentially causing deregulation of the AP of complement. A study conducted in 500 IgAN patients confirmed a possible role of CHFR5 role in IgAN, with higher soluble CFHR5 levels being associated with an increased risk of IgAN progression (Zhai et al., 2016; Zhu et al., 2018). Moreover, progressive IgAN has been associated with an increased glomerular CFHR5 deposition (Medjeral-Thomas et al., 2017). In 2010, a monoallelic (heterozygous) variant characterized by a two-exon duplication in the *CFHR5* gene was described in patients of Cypriot origin with familial glomerulonephritis (Gale et al., 2010). This “CFHR5 nephropathy,” which is morphologically a C3 glomerulopathy (C3G), shares a similar clinical course with IgAN, with persistent microscopic hematuria and progressive ESKD, but without the pathognomonic IgA mesangial deposits. This Cypriot *CFHR5* variant predicts an abnormally large protein with duplicated dimerization domains. The pathogenesis is thought to be secondary to the hetero-dimerization competing with the AP inhibitory activity of CFH (Goicoechea de Jorge et al., 2013). However, as Gale and Maxwell have commented, the link between *CFHR5* variants and C3G is not fully understood. The two most likely possibilities are a reduction in the effective concentration of active CFHR5 protein, or that the mutant CFHR5 protein acts in a dominant negative manner (Gale and Maxwell, 2013).

In 2012, Vernon et al. have reported on a 7-year old girl with “persistent post-infectious glomerulonephritis” who had a monoallelic (heterozygous) *CFHR5* variant (Vernon et al., 2012) (p.Glu163Argfs^*^34), that is very similar to the one observed in our patient (p.Gln121Lysfs^*^10). That variant, which in gnomAD is ~40 times more frequent than the variant of our patient, was associated with reduced serum CFHR5 levels. Interestingly, the variant was also present in her sister and mother, who were clinically healthy and who had a normal level of the CFHR5 protein (Vernon et al., 2012).

The subject of our study carries a CFHR1^*^B allele, that determines a slightly higher risk of developing aHUS compared with the CFHR1^*^A allele (Nakamura et al., 2018). However, this haplotype is extremely common (~30–40% of the general population) and cannot be considered pathogenic *per se*. In contrast, the *CFHR5* variant identified in our case is very rare (see above). By analogy to the variant observed by Vernon et al. it can be predicted that this variant results in partial CFHR5 deficiency. The circumstantial evidence of the role of similar *CFHR5* variants in different complement-mediated nephropathies suggests its pathogenic potential. However, this variant can be present, although very rarely, among healthy individuals. To summarize the evidence from genetic epidemiology, our observation seems to support and extend previous reports on the role of genetic variants of complement in the rapid progression of some cases of IgAN. However, the rare observation of these variants in healthy individuals indicates that no single variant so far seems to be sufficient to induce renal disease *per se* at the heterozygous state. The variants may increase the predisposition to develop the glomerulopathy or they could exacerbate the course of the disease (Kaartinen et al., 2019). The disease itself is likely triggered by other factors. The fact that *CFHR5* variants have been observed in association with different types of nephropathies (namely, C3G, persistent post-infectious GN, HUS, and –as in this case- IgAN) further supports the concept that they may contribute to glomerular disease rather than triggering it.

In view of the potential pathogenic consequences of the patient's genetic profile, we administered eculizumab up to 1 year post-transplant in order to prevent recurrence of the IgAN. Eculizumab, a humanized monoclonal antibody directed against C5, is the treatment of choice of aHUS, and has been employed in some cases of rapidly progressive IgAN associated with aHUS and *CFH* variant. In our patient, it was discontinued after 1 year post-transplant, because the genetic risk profile of the patient did not justify this long-term costly treatment and because its role in avoiding the recurrence of IgAN has not been established.

In summary, we report a case of IgAN associated with CFH-H3, MCP-H2, CFHR1^*^B polymorphisms, known to constitute moderate risk factors for aHUS, together with a very rare and deleterious *CFHR5* gene variant. This finding prompted us to use eculizumab, in parallel with the usual induction and maintenance transplant treatment. Over 3 years post-transplant the patient does not show clinical recurrence of IgAN. Biological and genetic screening of complement is currently part of the aHUS diagnostic process. We suggest that it should also be considered in rapidly progressive IgAN cases, to determine more precisely the prevalence of genetic and/or acquired complement abnormality, as this may have therapeutic implications in the future (Harris et al., 2018).

## Ethics Statement

Written informed consent was obtained from the patient for the publication of any potentially identifiable images or data included in this article.

## Author Contributions

All authors listed have made a substantial, direct and intellectual contribution to the work, and approved it for publication.

## Conflict of Interest

The authors declare that the research was conducted in the absence of any commercial or financial relationships that could be construed as a potential conflict of interest.

## Nomenclature

aHUS: atypical haemolytic uremic syndrome

AP: alternative pathway

CIC: circulating immune complexes

CFH: complement Factor H

CFHR5: CFH-related 5

IgAN: IgA nephropathy

CFHRs: complement FH related proteins (CFHRs)

ESKD: end-stage kidney disease

Gd-IgA1: galactose-deficient IgA1

GWAS: genome-wide association studies

LP: lectin pathway

TMA: thrombotic microangiopathy
